# Mission-oriented agrifood innovation systems in the making: a transdisciplinary approach to identify context-specific drivers of change

**DOI:** 10.1007/s11625-025-01719-2

**Published:** 2025-07-09

**Authors:** Alexandra Frangenheim, Marie Louise Schneider, Cornelia Fischer, Susanne Waiblinger, Stefan Hörtenhuber, Verena Radinger-Peer, Marianne Penker

**Affiliations:** 1 Department of Economics and Social Sciences, Institute of Sustainable Economic Development, University of Natural Resources and Life Sciences, Vienna, Feistmantelstraße 4, 1180 Vienna, Austria; 2 Department for Farm Animals and Veterinary Public Health, Institute of Animal Welfare Science, University of Veterinary Medicine Vienna, Veterinärplatz 1, 1210 Vienna, Austria; 3 Division of Livestock Sciences, University of Natural Resources and Life Sciences, Vienna, Gregor-Mendel-Straße 33, 1180 Vienna, Austria; 4 Institute of Landscape Development, Recreation and Conservation Planning, University of Natural Resources and Life Sciences, Vienna, Peter-Jordan-Straße 65, 1180 Vienna, Austria

**Keywords:** Transdisciplinary mission arena, Agrifood innovation system transformation, Leverage points, Austria, Beef, Dairy

## Abstract

A new generation of transformative policies has recently led to conceptual advances that map and evaluate agrifood innovation dynamics that contribute to the fulfillment of a societal mission within clearly defined time frames. In the context of the “European Green Deal”, national policies are being implemented with varying levels of government ambition and rigor, which is not yet reflected in these concepts. This article provides a framework and a methodology to help multi- and transdisciplinary teams to operationalize the transformation of an agrifood innovation system (AIS) into a mission-oriented agrifood innovation system (MAIS) in the absence of clear policy goals and objectives, i.e., where policymakers do not provide for the development of a mission. Within the safe space of a transdisciplinary research project, researchers and practitioners aim to understand where to start a sustainability transformation by examining subsectoral, transformative and directable drivers of change (DoC) in Austrian beef and dairy. To gather and integrate knowledge from different scientific and societal perspectives on how to transform this specific subsector, they established and facilitated a policy-independent, transdisciplinary mission arena. Besides context-specific results related to Austrian beef and dairy supply, we discuss the contribution of our transdisciplinary research to identify transformative and directable DoC as leverage points for transforming an AIS into a MAIS. Moreover, we explore the potentials, challenges and limitations of a policy-independent, transdisciplinary mission arena to provide orientation in the absence of national sustainability goals and objectives. Our conclusion includes a brief outlook for further research.

**Supplementary Information:**

The online version contains supplementary material available at 10.1007/s11625-025-01719-2.

## Introduction

Under the umbrella of the “European Green Deal”, the “Farm to Fork Strategy” and the “Biodiversity Strategy” have been formulated to enable transformative change in agrifood innovation systems (AIS) by 2030 (European Commission 2020a, 2020b). Mission-oriented innovation policies (Mazzucato 2016; Kattel and Mazzucato 2018) inspired a new generation of transformative approaches. Alongside challenge-led (Raven and Walrave 2020), challenge-oriented (Tödtling et al. 2022) or dedicated innovation approaches (Pyka 2017; Schlaile et al. 2022), the mission-oriented innovation systems (MIS) framework (Hekkert et al. 2020; Elzinga et al. 2023; Wesseling and Meijerhof 2023) has been provided as a conceptual basis for mapping and assessing innovation dynamics that contribute to the fulfillment of a societal mission. MIS go beyond the claim that policy should target public investments, but also seek to coordinate the innovation efforts of a wider range of actors toward well-defined goals in pre-defined time frames (Hekkert et al. 2020).

To understand how AIS needs to be transformed to support directed transformative change that benefits society as a whole, rather than supporting any innovation, Klerkx and Begemann (2020) posed key questions about the *what, why, who, where and how* of ‘mission-oriented agricultural innovation systems’ (MAIS). In a later article, Klerkx et al. (2022) broadened the term to ‘mission-oriented agrifood innovation systems’, also considering later stages of supply chains, i.e., (interactions between) actors from farm to fork, which fits our conceptualization of a MAIS for this article.

EU missions remain to be implemented at the national level (Janssen et al. 2023), i.e., they need to be backed up by national innovation policies to support the transformation of a national AIS into a MAIS. In addition to the underlying multi-scalar complexity, states play different roles in supporting innovations that solve societal problems depending on their constitutional, historical and cultural characteristics (Borrás and Edler 2020). The MAIS framework has been operationalized and enacted in countries, such as New Zealand, Australia and the Netherlands (Begemann and Klerkx 2022; Fielke et al. 2023; Klerkx et al. 2022), whose policy actors provide well-defined national goals. However, mission directionality emerges from and is shaped by various actors beyond the state (Klerkx and Begemann 2020) in arenas of contestation and negotiation (Wesseling and Meijerhof 2023). If national policymakers shy away from formulating concrete goals, e.g., because of the complexity and ambition of EU missions (Larrue 2021), other actors in the AIS will nevertheless move in the direction set by the EU mission, or, as Janssen et al. (2023, p. 409) put it, “mission-inspired problem solving is already taking off without clarity on available instruments”. MAIS development initiated by non-state actors has not yet been well conceptualized.

Austrian policy in general (Larrue 2021) and agrifood policy in particular (Schermer 2015; Frangenheim 2022) have been comparatively reluctant to identify and prioritize innovative, more sustainable forms of production and consumption. Although we observe converging views on the problem, the solutions are highly wicked (Wanzenböck et al. 2020). Ministry responsibilities are fragmented and policy instruments are not well coordinated across sectors (Serger et al. 2023). An indicator for a neglected sense of responsibility and goal setting by the government in the field of climate change is the fact that the national formulation of the “European Climate Law” is overdue since January 2021 (Görg et al. 2023).

Prior to the formulation of a national mission and the operationalization and governance of efforts to support its implementation (Elzinga et al. 2023), the *pooling of knowledge to meet* (context-specific) *challenges* (Wanzenböck et al. 2020) and the *setting of priorities and goals as a means to overcome directional failures* (Weber and Rohracher 2012) are important first steps. We argue that in countries where policy actors do not provide clear sustainability goals, affected actors can start by jointly identifying leverage points, here called drivers of change (DoC) that are directable and transformative in terms of supporting MAIS development.

Directability and transformability are highly context specific (Petschel-Held et al. 2006). To identify effective DoC in a policy-independent research project, it was purposeful to focus our empirical analysis on a national subsector with significant socio-economic relevance in the Austrian AIS. Austria has an oversupply of beef and dairy products related to the historical, economic and socio-cultural importance of cattle for alpine farming, regional identity and tourism in areas dominated by alpine grasslands. Their production, processing, marketing and consumption in diverse geographical contexts offer much potential for studying the diversity of change.

The article has two aims. The first is to operationalize a national MAIS in the making by integrating a policy-independent, transdisciplinary mission arena that was formed and facilitated within the safe space of the research project COwLEARNING^1^ rather than in a government-initiated process. To understand the context-specific scope of action in a country without clear agrifood sustainability goals and objectives, we showcase an iterative research design that includes a scientific scoping review to identify analytically formulated DoC, and a transdisciplinary approach to rate these DoC according to their transformative power and directability. As a second aim, we explore the specificities of transdisciplinary interaction in mission arenas in fields with convergent views on the problem, but high uncertainty about possible solutions (Wanzenböck et al. 2020). Guided by our framework that emphasizes the identification of subsectoral drivers of change to transform a conventional AIS to a MAIS, we pose the following research questions:

How does a transdisciplinary research design support the identification of transformative and directable DoC as leverage points for transforming an AIS into a MAIS?

What are the potentials, challenges and limitations of a policy-independent, transdisciplinary mission arena co-established by practice actors and researchers to provide orientation in the absence of national sustainability goals and objectives?

In this article, we provide a framework for the transformation from a conventional AIS to a MAIS by means of effective DoC (“Drivers of change related to agrifood innovation system transformation”). We present a methodology to help multi- and transdisciplinary teams to operationalize this in a combination of normal and postnormal scientific approaches (“Transdisciplinary research design”). In “Problems, developments and drivers of change in Austrian beef and dairy supply”, we present our context-specific results and in “Discussion”, we provide a synthesis of the results in relation to our research questions. A concluding section provides a summary and brief suggestions for further research.

## Drivers of change related to agrifood innovation system transformation

Open-ended conventional AIS support economic growth, competitiveness, and food security through interactive learning, new uses of knowledge for social and economic change, a broad understanding of innovation, and a wide range of actors (Spielman and Birner 2008). Technologies, institutional settings, attitudes, political economy factors, infrastructure, and research and innovation priorities have been built to support existing agrifood activities and tend to resist change (Conti et al. 2021). Thus, transforming an AIS to a MAIS is not a simple and straightforward process (Klerkx and Begemann 2020; Kok et al. 2019). Mission-oriented approaches differ from conventional innovation systems in that they aim to map and assess innovation dynamics and to design intervention strategies that contribute to the achievement of well-defined goals summarized in a mission (Hekkert et al. 2020; Wesseling and Meijerhof 2023). Teleological MAIS thus target concrete, often cross-disciplinary and cross-sectoral problems and offer a desirable direction of change in defined future timeframes (Klerkx and Begemann 2020).

The implementation of EU missions must be understood in its multi-scalar complexity. The provision of top-down regulation and control from the EU through strategies under the “European Green Deal” limits the autonomy in designing and implementing national strategies (Martin et al. 2022). Besides potential conflicts between levels, complexity and ambition of EU missions may also discourage policymakers from formulating concrete goals (Larrue 2021). AIS—and we transfer this to our MAIS framework—consider all actors potentially “involved in the creation, diffusion, adaptation, and use of all types of knowledge relevant” to agrifood supply (Spielman and Birner 2008, p. 4). This includes not only actors engaged in beef and dairy supply, but also those from heterogenous agrifood subsectors, such as grain, vegetable or wine production, as well as highly processed alternatives. If non-state actors in the AIS move in the direction set by the EU, they contribute to the development of an overall MAIS and, at the aggregate level, they put pressure on governments to coordinate subsectoral and regional activities.

While the argument for focusing on DoC or leverage points to initiate sustainability transformations is widely recognized, much attention is given to *parameters and feedbacks* rather than on *system design or intent* (Abson et al. 2017). The recent shift in innovation policy paradigms from narrow economic to broader societal goals (Polt 2019) arguably calls for greater consideration of deep leverage points (Meadows 1999) that transform system design and intent (Abson et al. 2017), but can also be successfully directed by affected actors.

In contrast to categorical distinctions between shallow and deep change (Abson et al. 2017; Riechers et al. 2022), we opt for a gradient from low to high transformative potential (similar to Slater et al. 2022). DoC with a higher degree of transformative potential are likely to bring about deeper changes and more fundamental shifts in the way actors think, behave, and operate, while those with a lower degree of transformative potential may only lead to incremental changes, e.g. by optimizing existing structures.

Our considerations on the degree of directability go beyond the term ‘degree of controllability’ used by proponents of the Millenium Eco-System Assessments literature (Gitay et al. 2005). We introduce the term ‘directability’ because of its propinquity to ‘directionality’. On the one hand, we aim to identify DoC that can be effectively addressed by actors affected by sustainability problems (to influence the pace of change). On the other hand, the term is commonly used in mission-oriented approaches, which are distinguished from other types of policy, such as systemic or challenge-oriented policy, by their emphasis on ‘intentionality’ (JIIP et al. 2018).

**Fig. 1:**
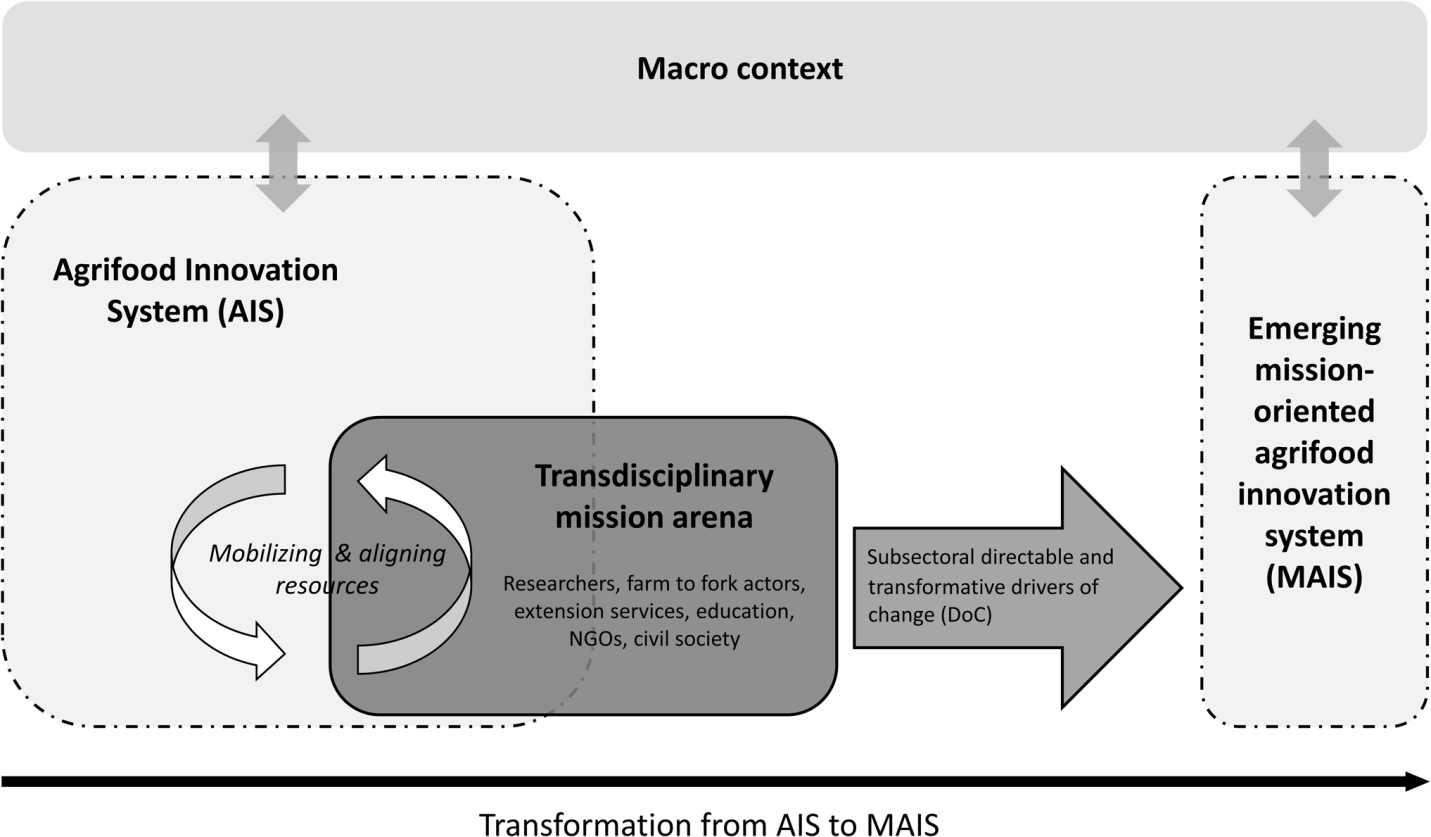
Relation between subsectoral drivers of change and agrifood innovation system transformation

Using the example of beef and dairy in Austria, we conceptualize the relation between subsectoral directable and transformative DoC, rated by a policy-independent, transdisciplinary mission arena, and the transformation from an open-ended national conventional AIS toward an MAIS. Focusing our analysis on dairy and beef as a key-subsector of the whole Austrian AIS allows for a context-specific consideration of actors’ needs and solutions and generates in-depth results from and for Austrian beef and dairy actors. Our framework, presented in Fig. 1, was developed primarily to guide our multi- and transdisciplinary analysis, but could also contribute to conceptualizations of AIS transformation.

While (M)AIS boundaries delimit directable actions and responsibilities to achieve innovation, the macro context is the environment outside of the system but coupled with its behavior (Ison 2017). According to de Boon et al. (2022), manifestations in the macro context result from changes in climate, biodiversity, demography, macroeconomics, and macropolitics (including transformative policy narratives of the “European Green Deal”) and influence the expectations and actions of AIS actors represented in the mission arena.

According to Wesseling and Meijerhof (2023), the transformation from a non-directed to a mission-oriented innovation system occurs through an *iterative mobilization and alignment of AIS structures into a semi-coherent ensemble* by actors that are represented in a mission arena. We illustrate that in the safe space of a research project, the policy-independent, transdisciplinary mission arena can imagine the transformation to a MAIS along transformative and directable DoC.

Inspired by Loorbach’s (2010) ‘transition arena’, the mission arena is a protected empowerment environment without formal hierarchy. Going beyond the transition arena’s focus on frontrunners—mostly involving proponents of the transition, the mission arena aims at the diversity of public and private actors involved in the transformation and representing different solutions with diverging societal benefits and sunk investments of the actors (Elzinga et al. 2023). In addition to the multidisciplinary research team, the mission arena involves established and innovative actors from farm to fork, extension services, education and value-based non-governmental organizations. Such a heterogenous mission arena makes it possible to build up new coalitions and partnerships, and to gather and integrate knowledge from different scientific and societal perspectives on how to transform the specific dairy and beef subsector. Operating without the direct support and involvement of national policy actors, the mission arena comes closest to Janssen et al.’s (2023) performance arena. This is defined as directly experiencing the outcomes of policy implementation, and is therefore best placed to explore its policy-independent leverage.

A DoC, as we understand it, affects the development of the subsector, in our case Austrian beef and dairy chains, from the breeding and rearing of animals, through the processing and trade of products, to their consumption in private and public settings. Some links in the supply chain may be more affected than others. However, a DoC always affects more than one link, if not all. DoC trigger context-specific changes in the past, present and future.

To support openness to actor-specific interpretations of how DoC could manifest in the future, they are formulated from an analytical and neutral perspective (e.g., change, as opposed to a normative way of growth or decline). In this way, a consensus on the most effective DoC can be reached without pre-determining DoC characteristics. Their actual direction of change then depends on the solution(s) preferred by each actor. For example, the DoC ‘knowledge and education’ related to agrifood may be manifested in a school subject and adult education programs, through privately initiated nutrition and consumer education in and out of school, or, it may be of limited importance in the education sector. Another example is the DoC ‘labels and production standards’, which may be interpreted by private and multi-dimensional initiatives, by EU climate and animal welfare standards, or by national legislation.

## Transdisciplinary research design

In 2020, the Austrian Science Fund launched the first call for “#ConnectingMinds” to support transformative research and the identification of societal solutions. With this call, the national funding organization for basic research provided a new way of funding, organizing and valuing research projects that open up to practice related knowledge (Rossing et al. 2021). The transdisciplinary team consists of researchers from innovation and agrifood transition studies, animal welfare science and sustainability assessment studies, as well as representatives of five cooperation partners, ranging from an animal welfare group that advocates for the rights of 1.8 million cattle in Austria, an association of 21.000 Austrian cattle breeders, a network that raises awareness of regional culinary products and the people behind them, an association that makes the functioning of agrifood chains transparent to consumers and an urban food policy council. Together, the team has set up, facilitated and is part of a project-specific mission arena with a total of 30 actors. The empirical database for this article was developed during the application phase and within the first year of the project.

**Fig. 2:**
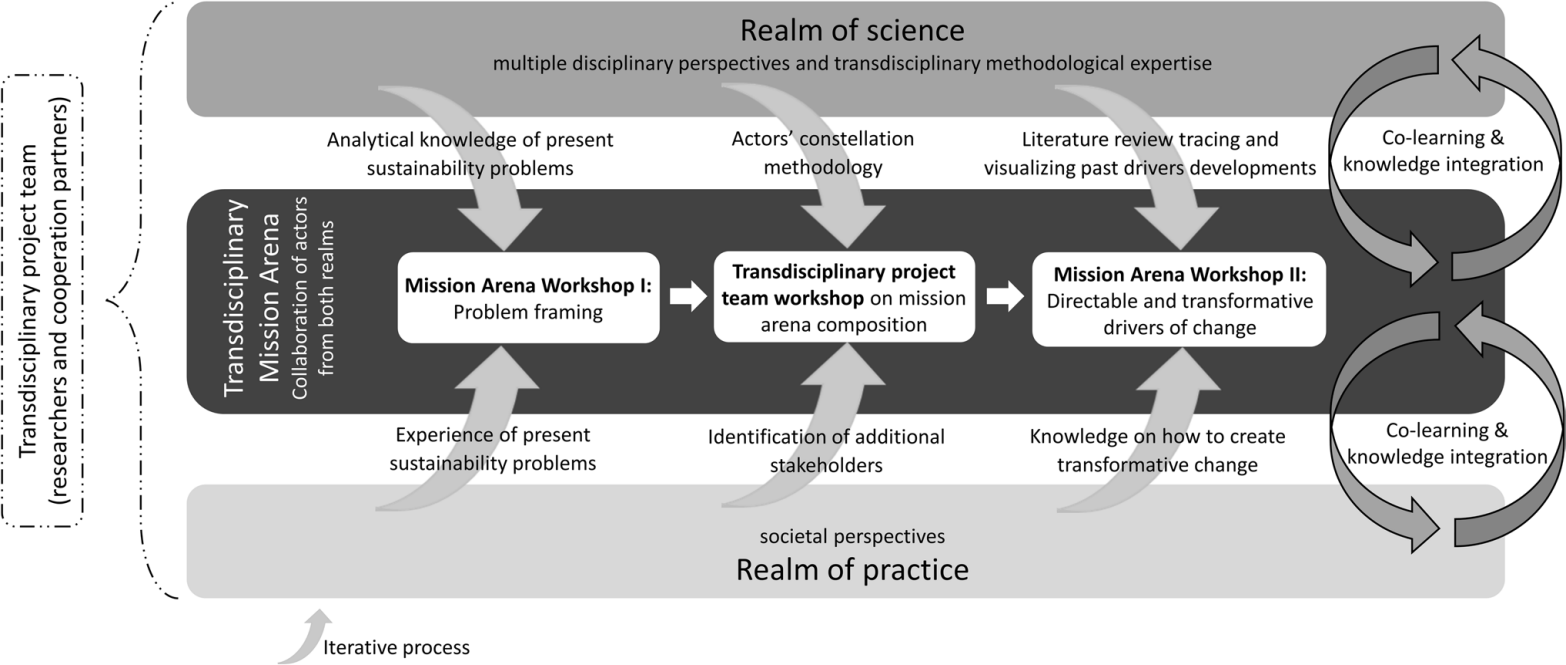
Iterative and transdisciplinary research process

The actors of the mission arena will co-develop scenarios, analyze and assess scalable sustainability innovations and transition pathways for the Austrian beef and dairy supply until 2027. To add knowledge on the early MAIS development phase in the absence of clear policy goals, we limit the analysis of this article to the iterative knowledge integration process (Lang et al. 2012) to identify effective DoC (Fig. 2). In the meetings with partners and other practice actors, we reflected in feedback rounds on the benefits and limitations of a transdisciplinary approach to establishing and facilitating a policy-independent mission arena.

Wesseling and Meijerhof (2023, p. 4) point out that the mission arena is “the central governance structure that mobilizes and redirects innovation system structures into a well-functioning M[A]IS”. While the realm of science consists of the multidisciplinary research team, the realm of practice is composed of the five cooperation partners and a group of collectively selected, invited and facilitated private and public representatives of the beef and dairy chain (feeding, breeding, farming, processing, marketing, gastronomy/public catering) and from related consulting firms, media organizations, a Chamber of Agriculture, educational institutions, NGOs and civil society.

Practitioners combine past experiences with expectations for the future through narratives to understand and communicate their interpretations of development processes (Steen 2016; Martin and Sunley 2022). To open up the view and consolidate knowledge within the mission arena, a narrative approach helped us to trace and visualize past DoC developments. In conducting a scoping literature review, we not only engaged academic experts in a participatory process (as in government-initiated mission development processes), but also drew on the written record of the last 70 years of scientific knowledge. By understanding historical developments as “an integral part of everyday sense-making and communicating” we transfer “interpretations and understandings of the past to how (actors) experience the present and set expectations for the future” (Wadhwani 2016, p. 67).

Such a process of historical contextualization begins with the identification of a need or problem in the present (Danto 1965 in Wadhwani 2016). The iterative research process therefore began with the context-sensitive problem framing developed in the first mission arena workshop (“Postnormal science: identifying drivers of change in future Austrian beef and dairy supply”; context-specific results see “Problem framing”). According to Wadhwani (2016), contextualizing the origins and historical development of contemporary sustainability problems that need to be addressed in the present or future is an analytical and interpretive endeavor that involves establishing relationships between events and their time and place. To explain causal and historical relationships, we therefore analysed past data from mono- or multidisciplinary ‘normal science’ (Riechers et al. 2022; methodology in “Normal science: identifying DoC in past Austrian beef and dairy supply”).

Between Workshop I and II, the transdisciplinary project team re-evaluated the composition of the mission arena. In a second mission arena workshop, we then confronted the actors with our list of DoC and asked them to complement, select and weight the 24 DoC and collectively decide on the most directable and transformative 16 DoC for the future (“Postnormal science: identifying drivers of change in future Austrian beef and dairy supply”; context-specific results see “Drivers of change in Austrian beef and dairy from farm to fork”). Identifying subsector-specific *current or future* effective DoC requires actionable knowledge that builds on “actors’ understanding of how to create transformative change towards sustainability” (Caniglia et al. 2021, p. 97). Deep leverage points are therefore identified by transdisciplinary ‘postnormal science’ with a more normative, solution-oriented focus (Riechers et al. 2022). The following two sections present our approach to combining normal and postnormal science.

### Normal science: identifying DoC in past Austrian beef and dairy supply

Based on the problem framing, the multidisciplinary research team conducted a scoping review (June to September 2022) of what has been studied about beef and dairy from farm to fork over the past 70 years from different disciplinary perspectives (Table 1). We focused on scientific review articles by authors affiliated with Austrian, German or Swiss research organizations, and contextualized our findings to the Austrian case by additionally searching for and analyzing documents and articles from federal authorities, specialized educational and research institutes, consultancies and NGOs (see Appendix for further details).

**Table 1:** Multidisciplinary data selection process and number of documents (research articles, book chapters, reports)

	Animal welfare science	Innovation and agrifood transition studies	Sustainability assessment studies
Review article from SCOPUS, Web of Science and CABI	640 documents	313 documents	212 documents
Included after first screening (peer-reviewed/grey literature)	197/0 documents	23/7 documents	22/0 documents
Additional peer-reviewed literature (focus on Austria)	6 documents	4 documents	6 documents
Additional grey literature (focus on Austria)	44 documents	8 documents	7 documents

We developed three search strings, i.e., one per scientific discipline, methodologically inspired by the PICO search strategy (Kuhn 2014) and followed the PRISMA review methodology (Moher et al. 2009; Page et al. 2021; see attachment 1). These search strings have been built based on existing knowledge of researchers and the problem framing developed during the first mission arena workshop (“Problem framing”). Table 1 provides an overview of the articles identified, screened and analyzed.

To identify DoC, we collected general information per article, such as the geographic scale considered, the time frame covered, specific milestones and actors/chain parts. Detailed information was collected on the socio-economic, environmental and animal welfare-related developments affecting humans and animals along beef and dairy chains, as well as their global and societal backgrounds. Based on this information, we developed the narrative and traced and visualized past DoC developments (context-specific results in “Past developments of directable and transformative drivers”).

We have deliberately included DoC that we consider to have a direct impact on the subsector or on the (M)AIS, as well as those that are more in the macro context but coupled with (M)AIS behavior, leaving their separation to the actors in the mission arena (cf. “Drivers of change in Austrian beef and dairy from farm to fork”). We first focused on the developments and corresponding DoC separately from the three disciplinary perspectives and then, in a multidisciplinary negotiation process, identified 24 DoC to take to the second mission arena workshop (cf. Table 2).

### Postnormal science: identifying drivers of change in future Austrian beef and dairy supply

We conceptualize the transdisciplinary mission arena workshops as spaces for experimentation (Schneidewind et al. 2016) or ‘safe spaces’ for co-learning, where participants can be confident that their statements will not be used against them, and that they will not suffer any disadvantages if they express critical or dissenting opinions (Bergold and Thomas 2012). Communication at eye level, ensured by appropriate methods and facilitation, stimulates knowledge integration, shared reflection and co-learning processes (Bergmann et al. 2010). Furthermore, friendly relationships, respect, the attractiveness of the group—the so-called ‘emotional dimensions’ (Jahn 2005; Pohl et al. 2021; Fischer et al. 2024)—are considered equally important conditions for co-learning (Godemann 2008).

Transdisciplinary research attaches great importance to the diversity of the actors involved (Enengel et al. 2012; Radinger-Peer et al. 2015), which increases creativity, salience and richness and reduces unintentional biases (Ernst et al. 2018). Accordingly, the main criteria for the selection of the 30 mission arena actors were to consider different knowledge types, topical expertise and experience, academic and non-academic backgrounds, public and private backgrounds, geographic diversity, gender, age and stake (Pohl and Hadorn 2007; Lang et al. 2012; Mitter et al. 2020).

Based on these criteria, the transdisciplinary team of researchers and cooperation partners compiled a list of actors relevant for beef and dairy in Austria who participated in the first mission arena workshop to inform the project design (November 2020). In a project team workshop (May 2022), the transdisciplinary project team evaluated this list according to its robustness/reliability and gaps (e.g., media) using an actor constellation (Frangenheim 2025). We assessed Austrian beef and dairy actors (e.g., farmers, gastronomy, extension service providers) regarding their power to influence the transformation of the subsector by slipping into their roles and positioning ourselves (1) near and far from a chair representing the problem and (2) in relation to each actor. After a self-assessment, everyone was allowed to give feedback on each other’s position, which revealed underlying assumptions and helped to identify the types of knowledge and expertise needed.

As the constellation of actual participants changed slightly in the two-day mission arena workshops I and II (cf. Janssen et al. 2023), participating organizations had the opportunity to send other representatives if the person originally nominated was unable to attend. Otherwise, the project team sought replacements, taking into account the actors’ role. The timing of the workshops (in the agricultural off-season) and the locations considered the accessibility for Austrian participants.

In the second mission arena workshop (September 2022), five heterogeneously composed groups of five to six participants were moderated by a heterogenous tandem of a researcher and a cooperation partner. We first completed the scientific analysis of past developments by identifying DoC that had been overlooked by the scientific realm (cf. “Drivers of change in Austrian beef and dairy from farm to fork”). The next step was to rate the DoC in terms of their capacity to bring about change in the future. In line with the “European Green Deal”, we imagined developments that could be expected over the next 30 years up to 2050. Thus, the identification of effective future DoC took into account the next generation and, overall, we have an observation period of 100 years. We asked two questions. First, ‘What are the transformative DoC within the 2050 timeframe?’ This refers to the extent to which a DoC has the potential to have a significant and lasting impact on individuals, organizations, or the AIS as a whole. Second, ‘Which DoC can the actors of mission arena jointly direct within the 2050 timeframe?’ This refers to the extent to which the actors can influence the pace and intention of a DoC (cf. “Drivers of change related to agrifood innovation system transformation”).

The outcome of each group was a pin board, on which the 24 DoC were positioned according to their directability and transformation potential ratings (see Table 2). To aggregate the group results, the number of groups that had rated the respective DoC in the upper half of both directability and transformation potential (upper right quadrant in the graphs of Table 2) was counted and the DoC were ranked accordingly. In a plenary discussion, we aggregated and discussed the group results and decided on the 16 most transformative and directable DoC.

## Problems, developments and drivers of change in Austrian beef and dairy supply

In this section, we briefly present the problem framing developed by the mission arena actors (“Problem framing”) and the results of the scoping review on past changes since 1950 on those 16 most directable and transformative DoC as rated by the mission arena actors (“Past developments of directable and transformative drivers”). We briefly present past DoC characteristics in narrative form, without discussing in detail the interactions, cumulative or obstructive effects of different DoC. “Drivers of change in Austrian beef and dairy from farm to fork” presents the directability and transformation potential of DoC in beef and dairy supply in Austria as rated by the transdisciplinary mission arena.

### Problem framing

The most important current challenges in Austrian beef and dairy are negative impacts on the climate and the environment, as well as on animal welfare in husbandry and slaughter, human living/working conditions and income, antibiotic use and resistance, health risks for humans and animals, price pressure, international competition and inequitable distribution of benefits among the actors from farm to fork, as well as an increased waste of meat and dairy products. In addition, polarization and a lack of mutual understanding among farm-to-fork actors create tensions around the following interests and objectives: the demand for cheap food, the lack of true cost pricing, the increasing need for trustworthy product information, resource-efficient structures and diverse societal demands, such as animal welfare, environment and health. These challenges and tensions are exacerbated by power imbalances, a lack of interaction between production and consumption and a loss of knowledge, skills and qualified personnel along the entire chain, which is then reflected in an uncertainty about what can be considered sustainable and what is not.

### Past developments of directable and transformative drivers

In line with the political ambition to provide affordable food to a growing population after the Second World War (Ermann et al. 2018), the majority of DoC have increased efficiency, (economic) growth and the implementation of technological solutions in beef and dairy from farm to fork. Overall, we observed a loss of small farms, less viable family farming, a change in the contribution of women, new methods of organization and cooperation, increased herd sizes and more intense, mechanized and specialized production, loss of biodiversity, concentration in processing and retailing and high consumption with lower relative expenditure on beef and dairy products.

‘Cattle breeding’ strategies combined with intensified feeding have increased performance per animal (e.g., doubling of milk yield in dairy cows over the past 50 years; Brito et al. 2021; ZAR 2020), and this trend appears to be continuing (Gross 2022). However, high performance places high demands on the animal’s metabolic system, often leading to a negative energy balance, distress and health problems (Bruckmaier and Gross 2017; Gross 2022).

The DoC ‘mechanization, specialization and concentration along chains based on the division of labor’, ‘legal and political requirements’, ‘development of costs and prices’, ‘changing lifestyles and dietary patterns’ and ‘image and status of work in agrifood industries’ supported efficiency gains and growth. They combined to produce a range of impacts on farm-to-fork actors and animals, which are discussed in the following three paragraphs.

On the side of cattle farming, specialization in beef and dairy production and the development of more ergonomic and labor-saving technologies (e.g., milking machines) and housing systems (e.g., slatted floors) enabled increases in herd size and intensification of yield, housing and feeding (Schön et al. 1991). These developments have been accompanied by a loss of genetic diversity in cattle and highly impaired welfare reflected in behavioral disturbances, high prevalence of production and housing-related diseases (e.g., mastitis, claw and joint lesions, ruminal acidosis), reduced longevity of dairy cows (Freyer et al. 2008; Humer et al. 2018; Punsmann et al. 2018; Oehm et al. 2019; Brito et al. 2021), and increased transport and mingling of calves associated with frequent use of antibiotics to control infectious diseases.

Socio-economic disadvantages include the marginalization of households in rural production areas, an image of precariousness, low qualification and wages, health problems in slaughterhouses, the replacement of traditional skills and ways of life, and the disappearance of cultural landscapes (Clay et al. 2020; Hostiou et al. 2020). On the other side, highly specialized production systems with an increased technology adoption have reduced farmers’ health burdens by replacing physical work with maintenance tasks (Hostiou et al. 2017).

On the environmental side, the drive for higher efficiency (e.g., higher yield per animal) has led to some environmental efficiency (e.g., reduced greenhouse gas emissions per kg product), but this has been accompanied by feeding more energy- and nutrient-dense diets, thus increasing feed–food competition (Ertl et al. 2015), as well as an intensification of feed production, leading to, among other things, loss of biodiversity (Allan et al. 2015). While forage was the sole component of cattle diets until the 1960s, today’s high-performance cows and fattening bulls are fed high amounts of grain- or corn-based concentrates, which pose challenges for ruminant digestion and behavior, resulting in health and behavioral disorders (Bruckmaier and Gross 2017; Humer et al. 2018), as well as for resource use and food security (Ertl et al. 2015). In addition, pasture-based husbandry systems have been replaced by zero-grazing systems, which allow for greater control of feed intake (Van den Pol-van Dasselaar et al. 2008), accompanied by further restrictions on cattle welfare (Hund et al. 2019).

A counteracting DoC is the ‘demand for cultural landscape with grazing’, which supports the (otherwise not economic) use of (alpine) pastures. Cattle play an important role in sustainable grassland management, which has received increasing interest in recent years (European Parliament and Council 2013) due to its relation to greenhouse gas fixation and its contribution to biodiversity by preventing grassland succession (Kiefer et al. 2020). Austria is one of the European countries with naturally large areas of grassland, and the landscape shaped by livestock (e.g., alpine pastures) is an immanent feature of regions with high tourist importance.

Manifestations of the DoC ‘shifting power relations in chains’ and ‘brands, labels and production standards (on the part of business in addition to legal regulations)’ have supported the increase in agrifood production units, the concentration of market power in large retail companies and the consolidation and industrialization of dairy and meat processing (De Schutter 2017; Penker et al. 2023). Dairies and retailers increasingly control the prices, cutting and packaging of beef products and define today’s ‘good’ animal husbandry systems and desirable animal traits, leading to reduced trust and understanding between the different parts of the chain (Hocquette et al. 2018). Large supermarket chains and industrial food producers, but also NGOs have created private brands that specify animal welfare and environmental standards (often in addition to legal regulations), thus defining production methods, the products available and what consumers know about them (Douphrate et al. 2013; Velázquez et al. 2017; Pröll et al. 2022).

Furthermore, there is increasing ‘pressure on agricultural land from industrial land use, transportation and settlement’ (Deblitz 2004). In the context of competitive land markets and structural change, fewer dairy and beef producers sought to exploit ‘economies of scale’, while ‘diversification’ strategies and ‘product and process innovation’ were less important (de Roest et al. 2018). Similarly, in commercial and industrial production and processing, ‘knowledge and education’ tended to promote efficiency rather than sufficiency and resilience (Clay et al. 2020; Schütt-Sayed 2020).

Internationally, milk and meat markets are also increasingly under pressure from (plant-based) ‘alternatives’, which have attracted significant investment from venture capitalists concentrated in Silicon Valley and in richer parts of Israel, Australia and Europe (Bojovic and McGregor 2022; Mason-D’Croz et al. 2022). Increased interest in ‘milk and meat alternatives’ also reflects ‘knowledge and education’ and the ‘reinterpretation of product and process quality’. There has been a shift in attitudes toward animals and animal products (Hocquette et al. 2018). While the importance of animal welfare was not recognized by the vast majority until the 1960s (Duncan 2019), European citizens now expect farmers to ensure the welfare of their livestock right after providing safe, healthy and sustainable food (European Commission 2022a) and believe it is important to protect the welfare of farmed animals (European Commission 2016). Welfare and sustainability labels introduced by retailers (cf. above) respond to the resulting consumer demands. However, existing ‘brands, labels and production standards’ do not necessarily benefit animal welfare, but rather contribute to distortions of competition and support the ‘renationalization’ of the EU market (European Commission 2022b). 

The developments described in the literature and above have been causing major welfare and environmental problems for several decades (Waiblinger 2009; Bojovic and McGregor 2022). Only recently have we seen—mostly international—incentives to transform the AIS to a MAIS, reflected in ‘legal and political requirements’ (e.g., the “Treaty of Amsterdam 1999”, the “UN Sustainable Development Goals”, “Food 2030”, the “European Green Deal” with the “Farm to Fork Strategy” and the “Biodiversity Strategy for 2030”, and the “Austrian Animal Welfare Act (Tierschutzgesetz) 2017”).

### Drivers of change in Austrian beef and dairy from farm to fork

Participants in the second mission arena workshop identified one DoC that has been overlooked in scientific analyses despite its potential impact on future change processes, namely ‘social media’. In addition, the DoC ‘knowledge, education and advertising’ was reorganized into ‘knowledge and education’ and ‘social media and advertising’.

Despite the variability in DoC ratings, the groups showed relatively good agreement (Table 2) on at least one of the two dimensions being in the lower or upper half (i.e., position in the same half of one dimension) in 14 of the 24 DoC. In three of these (knowledge, labels, costs) they agreed on both dimensions (i.e., position in the same quadrant). When the number of agreeing groups was reduced to four, six more DoC were located in the same quadrant and all but one (veterinary programs) were located in one half. Groups that deviated from the majority position varied randomly.

**Table 2:**
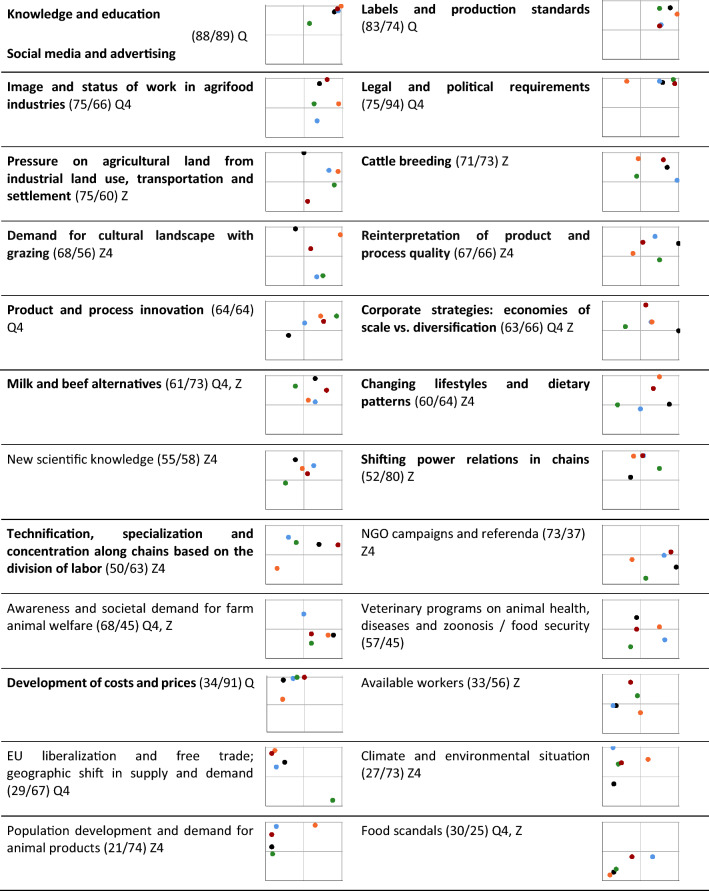
Directability (*D*, *x*-axis) and transformation potential (TP, *y*-axis) of the 24 drivers of change as rated by the mission arena

Drivers in bold were selected by the mission arena as the most directable and transformative. The original driver ‘knowledge, education and advertising’ was rearranged in the discussion following the rating session. Each graph shows the position of each driver as assigned in the group work; each dot represents one of the five groups. Drivers’ positions were measured as the relative distance to the end points (assigned 0 at the intercept of the *X* and *Y* axes for the lowest and 100 at the end of the axis for the highest *D* or TP). Thus, the upper right quadrant reflects drivers with relatively higher *D* and TP. Numbers in parentheses indicate mean relative *D*/TP. Drivers are ordered by mean *D*/TP (order: first both dimensions mean ≥ 50, then D ≥ 50 and TP < 50, then TP ≥ 50 and D < 50, then both < 50); Q: all 5 groups same quadrant; Q4: 4 groups same quadrant; Z: all 5 groups same half in 1 dimension, Z4: 4 groups same half

## Discussion

This section serves to discuss the two research questions: How does a transdisciplinary research design help to identify transformative and directable DoC as leverage points for transforming an AIS into a MAIS (“Operationalization of an emerging MAIS”), and what are the potentials, challenges and limitations of a policy-independent, transdisciplinary mission arena co-established by practice actors and researchers to provide orientation in the absence of national sustainability goals and objectives (“Potentials, challenges and limitations of transdisciplinary mission arenas”)? 

### Operationalization of an emerging MAIS

We have argued that in the context of shifting innovation policies that claim to address systemic, cross-disciplinary and cross-sectoral problems, the identification of directable and transformative DoC is an important precondition for understanding the scope of action in transforming an AIS into a MAIS. The combination of causal explanation (analysis of past data using mono- or multidisciplinary ‘normal science’) with goal-oriented, purposeful transformative research in transdisciplinary research designs (‘postnormal science’) has been identified as a key strength of a leverage point perspective (Riechers et al. 2022). However, existing leverage point perspectives (1) have generally been studied at a higher level of abstraction and from an external perspective (Meadows 1999; Abson et al. 2017; Chan et al. 2020), (2) are imprecise in terms of the perspectives and viewpoints of different actors and (3) do not provide an indication of the time frame over which they are effective. Comparing our context-specific and long-term ratings on the transformative potential and directability of DoC with Donella Meadows’ much-discussed perspective on shallow and deep leverage points (1999), we observe an important discrepancy that we cautiously discuss based on our operational design.

Our mission arena actors rated many of the drivers that Meadows classifies as less transformative but highly controllable, such as subsidies, taxes and standards, as less directable than transformative (‘legal and political requirements’, ‘development of costs and prices’, ‘available workers’ and ‘EU liberalization’). In part, this is because we have not involved policymakers. According to Meadows, leverage points are either easy to implement but have low transformative potential, or they are more difficult to alter but potentially lead to high transformative change. This dichotomy seems overly generalized. In our transdisciplinary research design, we therefore examined DoC for both their transformative potential and their directability, such as ‘knowledge and education’ and weeded out those with low directability and low transformative potential, such as ‘food scandals’.

Instead of identifying DoC at a higher level of abstraction and from an external perspective (Meadows 1999; Abson et al. 2017; Chan et al. 2020), we took an internal perspective of directly affected actors and their potential to contribute to the AIS transformation. This allows us to narrow down the wickedness of the solution and provide actors with a specific scope of action that is transformative, but still in their hands. Deliberately including DoC that we consider to have a direct impact on the subsector or on the (M)AIS, as well as those that are more in the macro context, we left the assignment to the actors in the mission arena. Some DoC that could be considered contextual to the subsector, such as ‘pressure on agricultural land from other uses’ and ‘milk and meat alternatives’ were nevertheless rated as being directable. Contrary to existing leverage point perspectives, our transdisciplinary design pointed out the need to consider competition or synergy with other economic (sub)sectors when transforming innovation systems (see also, e.g., Sandén and Hillman 2011; Frangenheim 2022). Conversely, the DoC ‘climate and environmental situation’ and ‘EU liberalization; geographic shift in supply and demand’ are rated very transformative, but hardly directable. To increase the directability of these very transformative DoC, we suggest that the interaction between humans and nature (Abson et al. 2017) and the obstruction or support of objectives and goals at different scales and in different countries (Trippl et al. 2024) should receive more attention in analyses of AIS toward MAIS transformation.

Moreover, our approach recognizes that different actors have different agency to intervene in a system, which makes a generalized understanding of effective DoC impossible. This is especially true when comparing practice actors with policymakers (Riechers et al. 2022). Transdisciplinary approaches do not assume that practitioners hold more or better knowledge than academics or policymakers, only different insights, experiences and preferences. While a common, concrete and tangible outcome can be facilitated in group work (“Potentials, challenges and limitations of transdisciplinary mission arenas”), the context-specificity of results makes it difficult to generalize DoC and transfer them to other subsectors.

Finally, although there is no direct indication of an appropriate time frame for leverage points (Meadows 1999; Abson et al. 2017; Chan et al. 2020; Riechers et al. 2022), we perceived the long-term perspective until 2050 as appropriate to identify those DoC that initiate AIS transformation and are in the hands of practice actors. Such a long time frame arguably creates the conditions to consider a broader range of DoC without determining their characteristics. For example, while lifestyles or work images may resist short-term change, they change over 28 years. Such consumer or demand perspectives have not been sufficiently considered in previous frameworks.

### Potentials, challenges and limitations of transdisciplinary mission arenas

Government-initiated mission arenas involve not only the mobilization and alignment of AIS resources, but also the renegotiation of support for traditional economic activities (Trippl et al. 2020). This points to power imbalances and tensions, particularly in an uncertain and wicked solution space (Wanzenböck et al. 2020). A mission arena that is initiated by a research process, has no direct political influence and therefore does not create winners and losers by initiating new legal requirements, funding or infrastructure. Instead, such a mission arena engages in a co-learning process, gaining insights into each other’s future expectations and strategies and thus learning about its own scope of action. A transdisciplinary mission arena can thus be seen as a catalyst that provides orientation in a situation where an EU mission is not (yet) implemented at the national level. This offers potentials, but also challenges.

The safe space of the research project focused on a subsector served as a field of experimentation for an emerging national MAIS. Participants in the workshop showed a genuine interest in collaboration and exchange, acting on their own initiative rather than being sent by a higher authority. It also helped to redress power imbalances, fostered a sense of equality and allowed for freedom of expression without prejudice. The project team was able to address important issues in the workshops independently, without being dictated to by higher-level institutions.

The joint selection of actors and the preparation and implementation of the workshops by the transdisciplinary team promoted the exchange of knowledge and the broadening of perspectives and problem analyses within the group (Godemann 2008). It also helped to balance power relations, enabled mutual appreciation, and signaled a willingness to share knowledge (Geels and Raven 2006; Van Mierlo and Beers 2020; Gugerell et al. 2023). A climate of trust and openness gives participants in a transdisciplinary research project the confidence that critical comments will not lead to disadvantages (Bergold and Thomas 2012), and an atmosphere of appreciation and emotional engagement (Jahn 2005; Pohl et al. 2021; Fischer et al. 2024) promotes the working atmosphere, encourages actors to openly share their thoughts and opinions and facilitates discussion. In group work, it was particularly up to the tandem of facilitators (from practice and science) to ensure the full involvement and decision-making power of all participants, although this requires careful preparation, if possible through prior test runs.

On the downside, however, the limited influence on the development of policy goals and direction made it difficult to engage mainstream actors (particularly from the hospitality and retail industries). In some cases we observed a self-selection of more innovative actors. This is consistent with Loorbachs’ (2010) ‘transition arena’ focus on frontrunners, but could limit the heterogeneity of perspectives during the workshops if not carefully considered.

Some context-specific, subsectoral challenges include different preferences regarding the duration of the workshop, and difficulties with the geographical diversity of actors’ origins due to travel times, which can be addressed by changing the location of successive workshops. Selection criteria that were relatively easy to meet were academic and non-academic background, gender and age (Mitter et al. 2019).

Of course, all the potential that transdisciplinary co-learning offers for addressing societal challenges cannot compensate for the limited strategic orientation of the government. Since the mission arena was established without an official mandate, it remains unclear what will happen to it at the end of the project in 2027, which ideas will be taken up, or whether commitment and funding will be available to continue it. Nevertheless, the project has already generated new insights, new networks and a farm-to-fork exchange between actors that did not exist before. It is to be expected that bottom-up initiatives like ours will collectively trigger political action toward MAIS development.

## Conclusions

The aim of this article is to provide a framework and a methodology to help multi- and transdisciplinary teams to operationalize the transformation of an AIS into a MAIS in the absence of clear policy goals and objectives. Based on a sound, interwoven process of a multidisciplinary scoping review of dairy and beef development over the past 70 years and a transdisciplinary process within the safe space of a research project, 16 transformative DoC were identified that can be directed by the actors of a policy-independent mission arena.

We chose Austria as a study site because its politicians are comparatively reluctant to formulate clear and accessible objectives and goals to support AIS transformation, making it a suitable case to study an emerging MAIS in a wicked solution environment. At the same time, the country is the reference area of the five-year transdisciplinary research project COwLEARNING, of which this research is part of, and which aims to provide orientation in this situation by creating a safe space and engaging diverse Austrian actors in a co-learning process on future sustainable beef and dairy supply in Austria.

The focus of our analysis on dairy and beef as a key subsector of the whole Austrian AIS led us to consider only a small part of the transformation from an AIS to a MAIS. However, this focus allowed for a more context-specific consideration of actors’ needs and solutions, and generated nuanced and in-depth insights from and for beef and dairy actors from farm to fork in Austria. Transferring the research design to other (sub)sectors in other (transnational, national or subnational) contexts with limited governmental involvement in establishing MAIS or uncertainty about how to stimulate MAIS would be necessary to test and refine the framework. Moreover, comparative cross-country case studies within Europe, but especially also beyond, would strengthen the empirical base across different institutional and political–economic contexts, and support deeper knowledge generation by identifying similarities and differences between multiple real-world examples and patterns across cases. After all, new transformative approaches, including mission-oriented innovation, are not only emerging from broadly defined EU missions, such as those under the “European Green Deal”. Transformation of AIS is called for globally, but visions of how to support and implement this differ in terms of goals and objectives, governance measures and solutions. 

## Supplementary Information

Below is the link to the electronic supplementary material.

Supplementary file1 (DOCX 25 KB)

## Data Availability

We provide information on DoC identification here in German: https://cowlearning.boku.ac.at/gemeinsam-identifizierte-treiber/.

## Footnotes

1 https://cowlearning.boku.ac.at/en/cowlearning-2/
