# Supplementary material for: Mission-oriented agrifood innovation systems in the making: a transdisciplinary approach to identify context-specific drivers of change
Source: Sustain Sci. 2025 Jul 9;20(6):2265–80. doi: 10.1007/s11625-025-01719-2 (PMC12554819; doi:10.1007/s11625-025-01719-2)
Supplement: Supplementary file 1 — Supplementary file1 (DOCX 25 KB) [file 11625_2025_1719_MOESM1_ESM.docx]

**Attachment 1: PICO literature search and scoping review methodology according to PRISMA**

To ensure an exhaustive and structured search approach, we developed three search strings (table below), i.e. one per disciplinary perspective (animal welfare science, innovation and agrifood transition studies, and sustainability assessment studies), methodologically inspired by the PICO search strategy (Kuhn 2014). While “Population” and “Intervention” terms differed per disciplinary perspective, terms for *Comparator* and *Outcome* were not specified in the search strings, which is a common approach in most qualitative research (Methley et al. 2014). While Population as well as Intervention terms are based on existing scientific knowledge of each discipline, *Intervention* terms have been additionally inspired by the problem framing developed during the mission arena workshop in December 2020 (section 4.1). Several trial searches enabled a refinement of search terms to ensure that most effective search terms were used.

We aimed at focusing on the Austrian context or contexts that are comparable to the Austrian socio-economic, cultural, physio-geographical, institutional and political structures and therefore limited the search on the *socio-economic situation* *of farm to fork actors* and *animal welfare* to review articles from Austrian, German or Swiss authors. To stay abreast of current research that claims for smaller-scale considerations of the *environmental situation*, we limited this search to studies about Austria without limiting to review articles. Instead, we used terms describing the development, change or driving forces to discover the effects of dynamic emission changes, site related combinations of soil, climate and related trends over time. To consider the global societal background, no geographical limitation has been applied.

Acknowledging that science about agricultural structures and more specifically about beef and dairy from farm to fork is an interdisciplinary endeavor (Mann 2021), we nevertheless streamlined the results by limiting to fields of studies of which the researchers have expertise on. Differences in the methodological development of the search strings have been discussed and carefully justified. Finally, to ensure the consideration of relevant case studies and empirical analyses about the development and drivers of change in beef and dairy from farm to fork specifically related to Austria, we included, based on previous knowledge and a web search, additional peer-reviewed publications and grey literature of federal institutes, specialized educational and research institutes, degree theses and relevant book chapters.

The scoping review followed the methodology of the PRISMA 2020 statement, performed by the same person that conducted the search (Moher et al. 2009; Page et al. 2021). After removing duplicates, we screened titles and abstracts of identified articles and assessed them according to their usability to provide information on developments and drivers of change for each disciplinary perspective. Articles that seemed useful to find information for the respective other disciplinary perspectives have been shared within the team and, if suitable, also screened by researchers from more than one perspective to integrate results and synthesize knowledge that is more than the sum of its disciplinary parts (Boix Mansilla 2006). The triangulation of data within the interdisciplinary research team not only ensured capturing every relevant study but also enhanced the validity of interpretations when selecting the long list of drivers of change in Austrian beef and dairy supply during the last 70 years.

**Table. Search string per scientific perspectives**

|  | **Animal welfare science** | **Innovation and agrifood transition studies** | **Sustainability assessment studies** |
| --- | --- | --- | --- |
| **POPULATION** | ***Animals:***  cow* OR cattle OR calf OR calves OR ox OR heifer* OR bull OR steer* OR bullocks OR “fattening bull*” OR “dairy cow*” | ***Beef/dairy actors from farm to fork:***  “cow farmer*” OR “cattle farmer*” OR “dairy farmer*” OR “slaughter house*” OR “dairy produc*” OR ((retail* OR logistic* OR restaurant OR gastronom* OR “public procurement of food” OR “public food procurement” OR “communal catering” OR consum*) AND (cow OR “dairy cow” OR cattle OR beef OR dairy OR milk OR cheese)) | ***Animals, products and inputs including cultivated areas:***  austria* AND (driv* OR chang* OR develop*) AND (cattle OR cow OR milk OR beef OR dairy OR feed OR roughage OR yield OR harvest) |
| **INTERVENTION** | ***Animal health, welfare and husbandry:***  housing OR “housing system*” OR husbandry OR “husbandry system*” OR environment OR stable OR tether* OR “loose housing” OR cubicle OR “free range” OR “outside yard” OR “outside run” OR pasture OR pen* OR herd OR floor OR flooring OR bedding OR litter OR “farming system” OR “space allowance” OR space OR enrichment OR “production system*” OR extensive OR intensive OR “industrial farming” OR breeding OR milk OR „milk yield“ OR milking OR beef OR „beef cattle“ OR production OR meat OR fertility OR veal OR mortality OR longevity OR lifespan OR performance OR “local breed*” OR “native breed*” OR  "animal welfare" OR welfare OR "well-being" OR wellbeing OR health OR disease OR “one health” OR “meat cattle welfare” OR “bovine welfare” OR “cow welfare” OR transport OR slaughter OR handling OR rearing OR “Cow-calf contact” OR “cow-calf-contact systems” OR “dam-bonded” OR “mother bonded” OR “milk feeding” OR weaning OR “Human-animal relationship” OR “Human-animal interaction*” OR behaviour OR behavior OR “normal behaviour” OR “normal behavior” OR “natural behaviour” OR “natural behavior” OR needs OR “welfare status” OR “social contact” OR horn OR horns OR horned OR hornless OR disbudding OR dehorning | ***Social and economic situation, global societal background, political and regulatory framework:***  WTO OR “trade agreement” OR EU OR “European Union” OR “EU regulation” OR “regulative framework” OR “EU regulative framework” OR policy OR “EU policy” OR “institutional change” OR governance OR “multi-level governance” OR “cultural shift” OR “societal change” OR societal transformation OR “industrial* agriculture” OR industrialization OR industrialisation OR globalization OR globalisation OR specialization OR specialisation OR rationalization OR rationalization OR digitalization OR digitalisation OR “structural change” OR “urban-rural dichotomy” OR “urban-rural binary” OR geography OR “technological change” OR automatization OR “productivity gains” OR robot* OR sensor* OR “just-in-time logistics” OR Innovat* OR resilien* OR competiti* OR “Human-animal relation*” OR (alienation AND animal) OR (alienation and cow) OR (alienation and cattle) OR “animal welfare” OR “animal welfare problems” OR consumption OR households OR “organic food” OR lifestyle OR “consumption behaviour” OR “consumption behavior” OR effects on health and wellbeing OR changing values OR “customer awareness” OR farm-to-fork OR Innovat* OR vegetarian* OR vegan* OR free-from products OR label* OR “designation of origin” OR “working conditions” OR “economic situation” OR “family life” OR “family income” OR “succession of farms” OR “farm succession” OR “land abandonment” | ***on- and off-farm environmental situation:***  "land cover" OR "land use" OR "nitrate" OR "phosphate" OR "greenhouse gas*" OR "ammonia" OR "acidification" OR "eutrophication" OR "energy demand*" |

# References

Boix Mansilla, Veronika. 2006. “Assessing Expert Interdisciplinary Work at the Frontier: An Empirical Exploration.” *Research Evaluation* 15(1):17–29. doi: 10.3152/147154406781776075.

Kuhn, Isla. 2014. *Systematic Literature Reviews- A “How to” Guide*. University of Cambridge Medical Library.

Mann, S. 2021. “Synthesizing Knowledge about Structural Change in Agriculture: The Integration of Disciplines and Aggregation Levels.” *AGRICULTURE-BASEL* 11(7). doi: 10.3390/agriculture11070601.

Methley, Abigail M., Stephen Campbell, Carolyn Chew-Graham, Rosalind McNally, and Sudeh Cheraghi-Sohi. 2014. “PICO, PICOS and SPIDER: A Comparison Study of Specificity and Sensitivity in Three Search Tools for Qualitative Systematic Reviews.” *BMC Health Services Research* 14(1):579. doi: 10.1186/s12913-014-0579-0.

Moher, D., A. Liberati, J. Tetzlaff, and D. G. Altman. 2009. “Preferred Reporting Items for Systematic Reviews and Meta-Analyses: The PRISMA Statement.” *Journal of Clinical Epidemiology* 62(10):1006–12. doi: 10.1016/j.jclinepi.2009.06.005.

Page, Matthew J., Joanne E. McKenzie, Patrick M. Bossuyt, Isabelle Boutron, Tammy C. Hoffmann, Cynthia D. Mulrow, Larissa Shamseer, Jennifer M. Tetzlaff, Elie A. Akl, Sue E. Brennan, Roger Chou, Julie Glanville, Jeremy M. Grimshaw, Asbjørn Hróbjartsson, Manoj M. Lalu, Tianjing Li, Elizabeth W. Loder, Evan Mayo-Wilson, Steve McDonald, Luke A. McGuinness, Lesley A. Stewart, James Thomas, Andrea C. Tricco, Vivian A. Welch, Penny Whiting, and David Moher. 2021. “The PRISMA 2020 Statement: An Updated Guideline for Reporting Systematic Reviews.” *Systematic Reviews* 10(1):89. doi: 10.1186/s13643-021-01626-4.
